# Electronic Structure and Minimal Models for Flat and Corrugated CuO Monolayers: An Ab Initio Study

**DOI:** 10.3390/ma16020658

**Published:** 2023-01-10

**Authors:** Anatoly A. Slobodchikov, Igor A. Nekrasov, Lyudmila V. Begunovich, Ilya A. Makarov, Maxim M. Korshunov, Sergey G. Ovchinnikov

**Affiliations:** 1 Institute of Electrophysics, Russian Academy of Sciences, Ural Branch, 620016 Yekaterinburg, Russia; 2 Federal Research Center KSC SB RAS, Akademgorodok, 660036 Krasnoyarsk, Russia; 3 Kirensky Institute of Physics, Federal Research Center KSC SB RAS, Akademgorodok, 660036 Krasnoyarsk, Russia

**Keywords:** CuO monolayer, band structure, DFT, minimal orbital model, Wannier functions projections

## Abstract

CuO atomic thin monolayer (mlCuO) was synthesized recently. Interest in the mlCuO is based on its close relation to ${\mathrm{CuO}}_{2}$ layers in typical high temperature cuprate superconductors. Here, we present the calculation of the band structure, the density of states and the Fermi surface of the flat mlCuO as well as the corrugated mlCuO within the density functional theory (DFT) in the generalized gradient approximation (GGA). In the flat mlCuO, the Cu-$3d_{x^{2}-y^{2}}$ band crosses the Fermi level, while the Cu-$3d_{xz,yz}$ hybridized band is located just below it. The corrugation leads to a significant shift of the Cu-$3d_{xz,yz}$ hybridized band down in energy and a degeneracy lifting for the Cu-$3d_{x^{2}-y^{2}}$ bands. Corrugated mlCuO is more energetically favorable than the flat one. In addition, we compared the electronic structure of the considered CuO monolayers with bulk CuO systems. We also investigated the influence of a crystal lattice strain (which might occur on some interfaces) on the electronic structure of both mlCuO and determined the critical strains of topological Lifshitz transitions. Finally, we proposed a number of different minimal models for the flat and the corrugated mlCuO using projections onto different Wannier functions basis sets and obtained the corresponding Hamiltonian matrix elements in a real space.

## 1. Introduction

Copper oxides stand apart from other transition metal compounds. First of all, they attract much attention because of their high temperature superconductivity (HTSC) and already existing applications [1,2,3,4,5] as catalysts [6], photocells [7] and thin-film transistors [8]. CuO is an exceptional member of the generally rocksalt family (MnO to CuO), as it deviates both structurally and electronically from others. Unlike other members of the 3d transition oxides, which crystallize in the cubic rocksalt structure (with possible rhombohedral distortions), Tenorite (CuO) crystallizes in the lower symmetry monoclinic (*C2/c*) crystal structure [9], albeit the cubic crystal structure is also possible [10]. Thus far, the bulk compound CuO has been thoroughly studied using ab initio calculations: DFT+U [11,12,13,14,15], DFT with hybrid functional [4] and Charge Transition Level Approach [16].

The relatively recent interest in the CuO monolayer arose in part because one would expect a superconducting phase to occur here by analogy with the typical representative of HTSC cuprates ${\mathrm{La}}_{2}{\mathrm{CuO}}_{4}$. There, the superconductivity occurs in two-dimensional layers formed by ${\mathrm{CuO}}_{2}$ plaquettes. The CuO monolayer consists of the same plaquettes. However, the plaquettes in the cuprates are connected by vertices, whereas in the monolayer they are connected by faces. This fact leads to a difference in the chemical composition—the number of copper and oxygen atoms is identical in the monolayer (CuO), while there are two oxygen atoms per copper atom in the cuprates (${\mathrm{CuO}}_{2}$).

In general, the electronic properties of the copper oxides are thoroughly studied. As everyone knows, ${\mathrm{La}}_{2}{\mathrm{CuO}}_{4}$ has the Cu-$d_{x^{2}-y^{2}}$ orbital at the Fermi level [5]. In Ref. [17] the authors studied isolated ${\mathrm{CuO}}_{2}$ monolayer using DFT and showed that all Cu orbitals, except for $d_{z^{2}}$, have states near the Fermi level; the Cu-$d_{xz}$ and $d_{yz}$ orbitals and the O-$p_{z}$ orbital have most of the states near the Fermi level, leading to π bonds in the entire monolayer. The bulk CuO with the monoclinic structure, known as a *p*-type semiconductor, has significantly three dimensional electronic structure with mainly Cu-3d (Cu-$3d_{x^{2}-y^{2}}$) states near the Fermi level [11]. DFT study in Ref. [14] claims that the bulk CuO with the cubic structure is an indirect gap semiconductor; its valence band consists mainly of O-2p and Cu-3d orbitals. We can conclude that the electronic structure of the ${\mathrm{CuO}}_{2}$ monolayer and ${\mathrm{La}}_{2}{\mathrm{CuO}}_{4}$ is quite similar to each other, while the one of the flat mlCuO partially resembles them, but has some qualitatively differences, such as an extremum presence in the Γ−M direction (as the reader can observe later).

**Figure 1:**
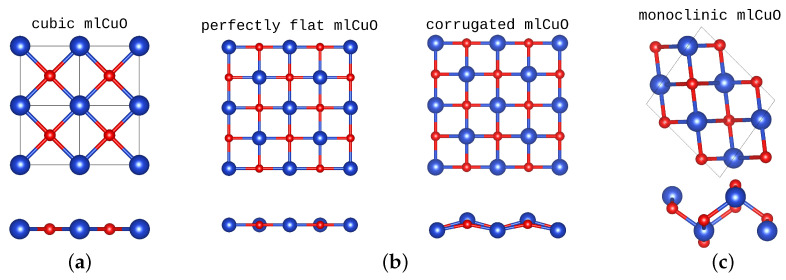
The mlCuO with the cubic (2×2×1 supercell) (**a**) and monoclinic (**c**) crystal structure, top and side view (following Ref. [14]). Freestanding perfectly flat and corrugated CuO monolayer crystal structure (**b**) (following Ref. [18]). Blue denote Cu atoms, red is O atoms. The figures are on the same spatial scale.

Moreover, a number of experiments and theoretical studies were carried out on various structural modifications of the CuO monolayer: CuO monolayer in a graphene pores and freestanding CuO monolayer [18], CuO monolayer on a graphene substrate [19], different combinations of CuO monolayers as an interface between bilayer graphene and finding thermodynamically stable freestanding CuO monolayer using the evolutionary algorithm [20]. In Ref. [18], the authors showed that freestanding the perfectly flat CuO monolayer can be corrugated in some cases (Figure 1b). This perfectly flat mlCuO can be easily constructed from the bulk CuO with the cubic structure. In Figure 1a, we show the 2×2×1 supercell of the cubic mlCuO. It matches with the perfectly flat mlCuO structure (Figure 1b, left) when rotated by $45^{\circ }$. The corrugated mlCuO can be constructed from the bulk CuO with the monoclinic structure, though in the monoclinic system atoms are much more displaced; see Figure 1c and compare it with Figure 1b, right. Thus, the corrugated state of mlCuO can be described as a transitional one relative to the CuO systems with the cubic and the monoclinic structures.

The description of the electronic structure of mlCuO systems is also far from being complete—the authors were able to find some data in Ref. [18,20], but no more than that. Besides, at the moment the authors are not aware of any works where a minimal model has been formulated neither for an isolated perfectly flat monolayer CuO, nor for more complex crystal structures such as the corrugated monolayer, monolayer on a substrate or monolayer as an interface. Thus, it seems necessary to obtain on a more systematic basis the densities of states, the band structures and the Fermi surfaces for all listed monolayer CuO systems and to formulate a minimal model for them with the corresponding Hamiltonian parameter values.

In this work, we solve a task of proposing and comparing different minimal models for the CuO monolayer systems as a necessary first step of any further theoretical investigations.

## 2. Crystal Structure and Calculation Details

To calculate the band structure, the density of states (DOS) and the Fermi surface, we used the density functional theory with the full-potential linear augmented plane-wave framework, as implemented in WIEN2k [21] together with the generalized gradient approximation by Perdew, Burke and Ernzerhof [22], to the exchange-correlation functional.

**Figure 2:**
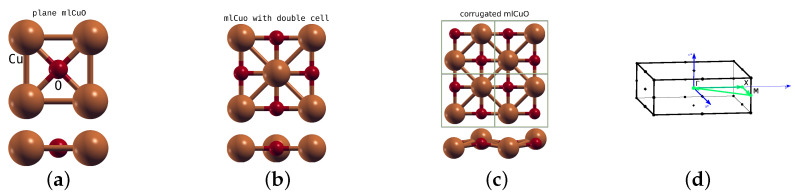
The flat mlCuO (**a**), the mlCuO with a doubled unit cell (**b**), the corrugated mlCuO (2×2×1 supercell) (**c**) crystal structures considered in this work, top and side view; brown denote Cu atoms, red is O atoms. Brillouin zone for the doubled unit cell and corrugated mlCuO (**d**).

Figure 2 shows the crystal structures of the systems discussed in this paper. The flat CuO monolayer space group is a 123 (*P4/mmm*). The lattice parameter is a=2.69 Å [18]. Atoms occupy the following positions: Cu 1a (0,0,0) and O 1c (0.5,0.5,0). We used a 20 Bohr vacuum gap. In order to construct the corrugated CuO monolayer, we doubled the unit cell and made these new additional Cu and O atoms unequivalent to the original ones by applying a small shift about 0.5 Bohr in *z* direction only for them. Resulting system has 59 (*Pmmn*) [origin choice 2] space group. Next, we did a set of structural relaxations with 10, 20 and 40 Bohr vacuum gaps. There was no difference between 20 and 40 Bohr vacuum gap cases, so in all further calculations we used the 20 Bohr gap. After relaxation neighboring Cu atoms shifted in *z* direction about ±0.31 Å relative to their original positions, while neighboring O atoms barely shifted at all. Since the final corrugated structure of CuO monolayer has a doubled unit cell and is rotated by $45^{\circ }$ relative to the flat mlCuO, we cannot directly compare their calculated electronic structures. Thus, we use additional system—the flat mlCuO with a doubled (and rotated) unit cell—in order to make a proper comparison. Moreover, for the flat mlCuO we did a $45^{\circ }$ rotation of a local coordinate system in order to use a typical orbital convention, such as in cuprate compounds.

All calculations were nonmagnetic and converged self-consistently on a grid of 24 × 24 × 1 *k*-points in the irreducible Brillouin zone using the Monkhorst-Pack method [23]. We used energy convergence limit 0.1 mRy, force convergence limit 0.5 mRy/a.u. for optimization, RKmax=7, Gmax=12, energy separation −6.0 Ry. In Figure 2d, we show the Brillouin zone with the *k*-path used in the band structure analysis.

## 3. Results and Discussion

### 3.1. Electronic Structure

**Figure 3:**
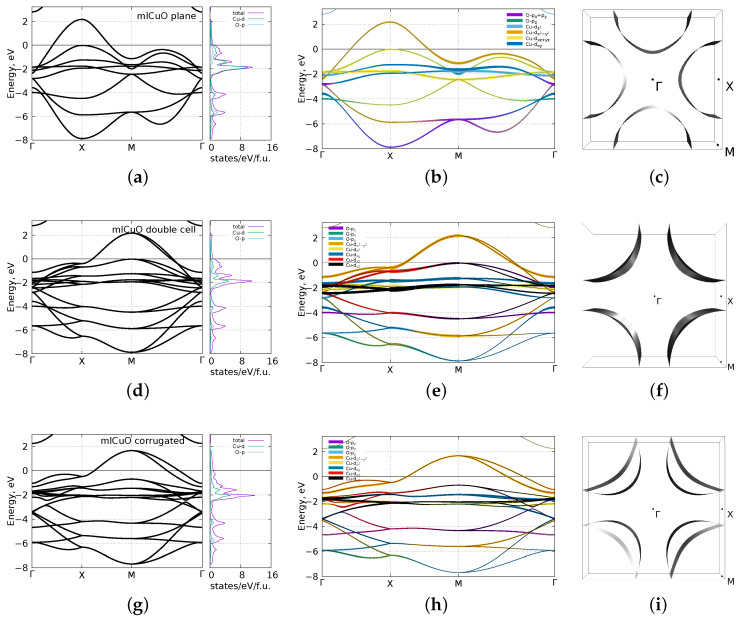
DFT (GGA) calculated DOS, the band structures, the band structures with the orbital characters and the Fermi surface of the flat mlCuO (**a**–**c**), the mlCuO with a doubled unit cell (**d**–**f**), the corrugated mlCuO (**g**–**i**). Zero corresponds to the Fermi level.

Figure 3 shows the DFT (GGA) band structures, the densities of states, bands with their orbital characters and the Fermi surfaces. The first row (a–c) of Figure 3 shows the results for the flat mlCuO. The band structure in Figure 3a shows that there is an isolated set of bands in the range from −8 eV to 2.3 eV resembling typical Cu-based HTSC ${\mathrm{La}}_{2}{\mathrm{CuO}}_{4}$. It has only the Cu-3d and the O-2p states. The electronic bands of the flat mlCuO at the Fermi level are formed by the Cu-$3d_{x^{2}-y^{2}}$ states (with small addition of the hybrid O-2p states) in consistence with the known results [11]. Note that there is a second band that almost crosses the Fermi level—it is only 0.02 eV lower. It includes the Cu-$3d_{xz,yz}$ states hybridized with the O-$2p_{z}$. The Fermi surface has a hole pocket around the *X* point.

The second row (d–f) of Figure 3 shows the results for the mlCuO with a doubled unit cell. Due to $45^{\circ }$ rotation of a unit cell, its band structure differs from that of the flat mlCuO in a much more complex way than simply by a large number of bands. The two bands crossing the Fermi level originate from two Cu atoms in the unit cell. They are formed by the Cu-$3d_{x^{2}-y^{2}}$ states. Regarding them, we can note degeneracy lifting in the Γ−M direction. As in the flat mlCuO, there are the Cu-$3d_{xz,yz}$ states (four bands) just below the Fermi level. The Fermi surface has two hole pockets around the *M* point.

The third row (g–i) of Figure 3 shows the results for the corrugated mlCuO. Its electronic structure is rather similar to that in the mlCuO with a doubled unit cell, but there are two notable differences. First, a significant shift of the Cu-$3d_{xz,yz}$ bands to −0.7 eV. Second, a noticeably larger degeneracy lifting for the Cu-$3d_{x^{2}-y^{2}}$ bands in the Γ−M direction. On top of that, the corrugated mlCuO total energy turns out to be lower than the flat mlCuO one by 0.07 eV. In other words, the corrugated state appears to be more favorable and if we have the flat mlCuO as a topmost layer of some surface, it will most likely be corrugated.

Seeing such a significant shift of the Cu-$3d_{xz,yz}$ bands of the corrugated mlCuO, we wondered if it was possible to raise these states to the Fermi level only via the lattice strain. Significant lattice strain is observed at all sorts of interfaces where there is a mismatch between the lattice parameters. Besides, as we already mention, the corrugated state appears to be more energetically favorable.

**Figure 4:**
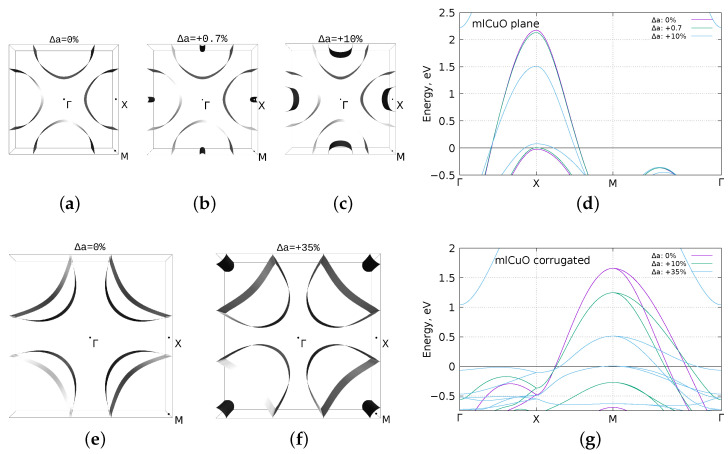
DFT (GGA) calculated Fermi surface with Δa=0% (zero strain), Δa=+0.7%, Δa=+10% and the band structure comparison for the flat mlCuO (**a**–**d**); the Fermi surface with Δa=0% (zero strain), Δa=+35% and the band structure comparison for the corrugated mlCuO (**e**–**g**). Zero corresponds to the Fermi level.

To clarify this issue, we carried out a series of calculations where we varied the lattice parameter *a* from 0% to 10% for the flat mlCuO and from 0% to 35% for the corrugated mlCuO. The corresponding results can be observed in Figure 4. For the flat mlCuO, the lattice deformation Δa=0.7% leads to a topological Lifshitz transition with the appearance of a new hole pocket around the *X* point. Clearly, it is a very minor lattice parameter change of the order of experiment accuracy. For the corrugated CuO monolayer, such a transition requires a much larger lattice deformation; it appears only at Δa=35%. Of course, such a strain is too large, and we bring it here only as an illustration. However, for the corrugated mlCuO case, we want to note a presence of what seems to be a flat band in the Γ−X direction near the Fermi level. It is likely that a flat band at the Fermi level can be obtained using a reasonable lattice strain and a hole doping.

Let us now return to the idea we proposed in the introduction: the corrugated of mlCuO can be described as a transitional system in between the CuO with the cubic and monoclinic structures (Figure 1). Table 1 shows Cu-Cu and Cu-O distances in the bulk CuO systems and the CuO monolayers. In the bulk CuO with the cubic structure, CuO layers are significantly stretched—by about 11.5% relative to the flat mlCuO, which is close to the considered lattice strain Δa=10% (Figure 4c). We studied the monolayer made from the bulk CuO by simply adding a vacuum between layers (getting slab+vacuum) in *z* direction and using no relaxation (Figure 1a). The final crystal and band structures were nearly identical to the flat mlCuO ones with lattice strain Δa=10% (as in Figure 4c,d); thus, we do not include them.

**Figure 5:**
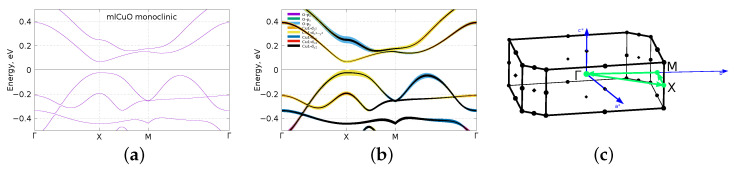
DFT (GGA) calculated band structure, the band structure with the orbital characters and the corresponding Brillouin zone (**a**–**c**) of the monoclinic mlCuO. Zero corresponds to the Fermi level.

There are more surprising results for the bulk CuO with the monoclinic structure. Its CuO layers are also stretched, but to a noticeably lesser extent—by about 5.1% relative to the corrugated mlCuO. Its crystal structure has more pronounced corrugation pattern as compared to the corrugated mlCuO (Figure 1c). To calculate the monoclinic CuO monolayer, we again made a slab+vacuum in *z* direction and used no relaxation; moreover, we did a $45^{\circ }$ rotation of a local coordinate system to remain consistency with the orbital convention chosen for the corrugated mlCuO. The final space group was 13 (*P2/c*). The results are given in Figure 5. The most surprising result is that the monoclinic mlCuO has the band gap at the Fermi level, which opens because of symmetry lowering without using DFT+U or hybrid potentials, as (but for the bulk compound) in Ref. [4,11]. There are mainly the Cu-$3d_{x^{2}-y^{2}}$ (with the hybrid O-2p) states near the Fermi level similar to the corrugated mlCuO. However, it is rather difficult to compare these results in detail with the results for the corrugated mlCuO (Figure 3g,h) due to the different space group. Let us conclude here that a more complex corrugation pattern leads to a more complex bands structure.

### 3.2. Minimal Models

**Figure 6:**
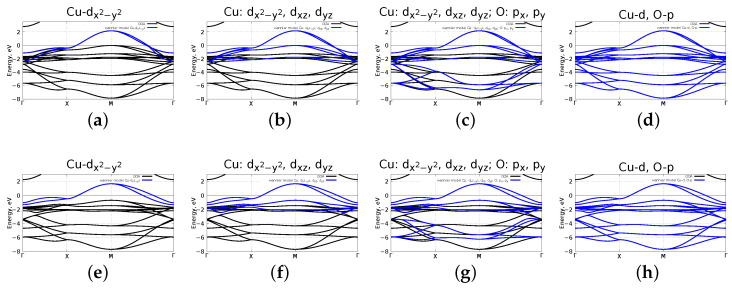
Comparison DFT (GGA) band structure with a Wannier projected one: Cu-$d_{x^{2}-y^{2}}$ (**a**), Cu-$d_{x^{2}-y^{2}},d_{xz},d_{yz}$ (**b**), Cu-$d_{x^{2}-y^{2}},d_{xz},d_{yz}$, O-$p_{x},p_{y}$ (**c**), Cu-*d*, O-*p* (**d**) for the mlCuO with a doubled unit cell; same for the corrugated mlCuO (**e**–**h**). Zero corresponds to the Fermi level.

The next step in our study is to reveal a good minimal model for both flat and corrugated mlCuO. In order to do that, we constructed a set of models using the maximally localised Wannier functions (MLWF) within wannier90 package [24]. We examined the following set of models: single-band model with Wannier projected Cu-$d_{x^{2}-y^{2}}$, three-band model with Cu-$d_{x^{2}-y^{2}},d_{xz},d_{yz}$, five-band model with Cu-$d_{x^{2}-y^{2}},d_{xz},d_{yz}$, O-$p_{x},p_{y}$ and eight-band model with Cu-*d*, O-*p*. The corresponding band structures are depicted in Figure 6. Note that there are actually twice the number of bands for the corrugated mlCuO and the mlCuO with a doubled unit cell due to unit cell doubling in contrast to the flat mlCuO. The eight-band model resulting band structure is in the excellent agreement with GGA calculated one; in other cases, the agreement is fine at the Fermi level. Thus, the single-band Cu-$d_{x^{2}-y^{2}}$ model for the flat and the corrugated mlCuO can be used as a minimal model.

**Figure 7:**
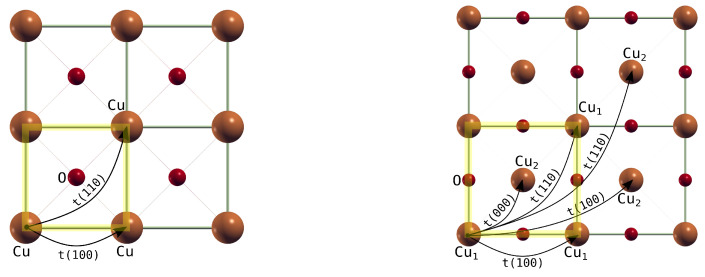
Hoppings schemes for the flat mlCuO (**left**) and the mlCuO with a doubled unit cell (**right**). A 2×2×1 supercell is displayed for both structures. Yellow transparent square denotes a single unit cell.

We also present on-site energies and hopping integrals for one- and three-band models for the flat (Table A1), the corrugated mlCuO and the mlCuO with a doubled unit cell (Table A2 and Table A3). The corresponding hopping schemes are illustrated in Figure 7. We used hoppings up to the second coordination sphere for the flat mlCuO and up to the fifth coordination sphere for the corrugated one. These numbers of neighbors are the minimum required to obtain good agreement between the model Hamiltonian band structure and the initial one. We also attach the values of the Hamiltonian matrix elements in a real space for all the models (Figure 6) as machine-readable data files to Supplementary Materials. So, depending on a task, an interested reader can use the Hamiltonian of the appropriate complexity.

## 4. Conclusions

We investigated the electronic properties of the flat and the corrugated CuO monolayers—DOS, the band structures and the Fermi surfaces. The flat mlCuO is similar to the bulk CuO with the monoclinic crystal structure and typical Cu-based HTSC, e.g., ${\mathrm{La}}_{2}{\mathrm{CuO}}_{4}$ and has the Cu-$3d_{x^{2}-y^{2}}$ states at the Fermi level (with a small addition of the hybrid O-2p states). There is a second band just below the Fermi level—that it is only 0.02 eV lower. It includes the Cu-$3d_{xz,yz}$ states hybridized with O-$2p_{z}$.

The corrugation effect leads to a significant shift of the Cu-$3d_{xz,yz}$ bands to −0.7 eV and a degeneracy lifting for the Cu-$3d_{x^{2}-y^{2}}$ bands. The corrugated mlCuO is energetically more favorable than the flat one by 0.07 eV and is more likely formed as a topmost layer on some surfaces.

It is possible to create a topological Lifshitz transition via lattice strain: for the flat mlCuO, a slight stretching of the lattice parameter Δa=0.7% already leads to the appearance of a hole pocket around the *X* point; this effect for the corrugated mlCuO occurs at Δa=35%. It is interesting to note the presence of what seems to be a rather flat band in the Γ−X direction near the Fermi level. Probably, it will be possible to create it using a reasonable strain and a hole doping.

There is a significant mismatch in the lattice parameters of the considered CuO monolayers and the known bulk CuO systems (with the cubic and the monoclinic crystal structures). We conclude that CuO layers in the bulk CuO are stretched relative to the monolayer systems—by about 11.5% (cubic bulk vs. flat monolayer) and by 5.1% (monoclinic bulk vs. corrugated monolayer). The monolayer made from experimental bulk CuO with the monoclinic crystal structure turns out to have the band gap in our DFT calculation without using DFT+U or hybrid potentials. Clearly, the reason for this is the more complex corrugation patterns of its crystal structure.

We also suggested a set of minimal models for the flat and the corrugated CuO monolayers. The simplest model includes only the Cu-$d_{x^{2}-y^{2}}$ states and agrees well with the GGA calculated band structure at the Fermi level (the latter is also correct for the other models). For the one- and three-band models, we obtained the values of the corresponding Hamiltonian matrix elements in a real space; therefore, depending on the needs, the Hamiltonian of the appropriate complexity can be used.

Before proceeding to more complex systems, such as a CuO monolayer on a substrate or a CuO monolayer as an interface, it was necessary to perform calculations for the original system and understand the features of its electronic structure. So, the next step might be the investigation of such complex systems or using more advanced methods such as DFT+DMFT.

## Figures and Tables

**Table 1:** Cu-Cu and Cu-O distances in the bulk and the monolayer CuO systems.

System	d(Cu-Cu), Å	d(Cu-O), Å	Ref.
Cubic bulk CuO	3.00	2.12	[10]
Monoclinic bulk CuO	2.90	1.96	[9]
Flat mlCuO	2.69	1.90	This work, ref. [18]
Corrugated mlCuO	2.76	1.93	This work

## Supplementary Materials

The following supporting information can be downloaded at: https://www.mdpi.com/article/10.3390/ma16020658/s1.

## Appendix A

We attach the values of the Hamiltonian matrix elements of all the models considered in this work as Supplementary Materials. These are machine-readable data files with self-explanatory names and some comments.

**Table A1:** An on-site energies and hoppings in meV for the flat mlCuO (one- and three-band models).

	Cu-$d_{x^{2}-y^{2}}$			Cu-$d_{x^{2}-y^{2},\mathrm{xz},\mathrm{yz}}$		
$m^{\prime },m$	ε(000)	t(100)	t(110)	ε(000)	t(100)	t(110)
$x^{2}-y^{2},x^{2}-y^{2}$	126	−69	−485	169	−43	−461
xz,xz				−1417	−227	−139
yz,yz				−1417	226	−139
xz,yz				0	0	−191

**Table A2:** An on-site energies and hoppings in meV for the doubled unit cell and corrugated mlCuO (one-band model).

	${\mathrm{Cu}}_{1,2}$-$d_{x^{2}-y^{2}}$					
	Doubled u.c.			Corrugated		
$m^{\prime },m$	ε(000)	t(100)	t(110)	ε(000)	t(100)	t(110)
$x^{2}-y_{\mathrm{Cu}}^{2}1,x^{2}-y_{\mathrm{Cu}}^{2}1$	124	−486	73	161	−351	86
$x^{2}-y_{\mathrm{Cu}}^{2}1,x^{2}-y_{\mathrm{Cu}}^{2}2$	−70	−43	13	5	−19	−19

**Table A3:** An on-site energies and hoppings in meV for the doubled unit cell and corrugated mlCuO (three-band model).

	${\mathrm{Cu}}_{1,2}$-$d_{x^{2}-y^{2},\mathrm{xz},\mathrm{yz}}$					
	Doubled u.c.			Corrugated		
$m^{\prime },m$	ε(000)	t(100)	t(110)	ε(000)	t(100)	t(110)
$x^{2}-y_{\mathrm{Cu}}^{2}1,x^{2}-y_{\mathrm{Cu}}^{2}1$	167	−462	82	24	−391	85
$x^{2}-y_{\mathrm{Cu}}^{2}1,x^{2}-y_{\mathrm{Cu}}^{2}2$	−44	−36	10	−39	−19	−19
$x^{2}-y_{\mathrm{Cu}}^{2}1,xz_{\mathrm{Cu}}1$	0	0	0	0	−278	30
$x^{2}-y_{\mathrm{Cu}}^{2}1,xz_{\mathrm{Cu}}2$	0	0	0	41	31	31
$x^{2}-y_{\mathrm{Cu}}^{2}2,xz_{\mathrm{Cu}}1$	0	0	0	41	−41	7
$x^{2}-y_{\mathrm{Cu}}^{2}1,yz_{\mathrm{Cu}}2$	0	0	0	−20	−34	34
$xz_{\mathrm{Cu}}1,xz_{\mathrm{Cu}}1$	−1417	−329	−8	−1430	−128	−40
$xz_{\mathrm{Cu}}1,xz_{\mathrm{Cu}}2$	18	41	5	0	14	14
$yz_{\mathrm{Cu}}1,yz_{\mathrm{Cu}}1$	−1417	53	−8	−1439	43	−3
$yz_{\mathrm{Cu}}1,yz_{\mathrm{Cu}}2$	18	−12	5	−6	4	4
$xz_{\mathrm{Cu}}1,yz_{\mathrm{Cu}}1$	0	0	47	0	0	28
$xz_{\mathrm{Cu}}1,yz_{\mathrm{Cu}}2$	−246	−21	−214	−206	2	−2
$xz_{\mathrm{Cu}}2,yz_{\mathrm{Cu}}1$	−246	246	−246	206	−206	−24
